# Studies in the Psychology of Sex

**Published:** 1904-02

**Authors:** 


### Books and Magazines

Studies in the Psychology of Sex. Analysis of the Sexual Impulse, Love and Pain; the Sexual Impulse in Women. By Havelock Ellis. 258 pages. Price, $2.00. Published by F. A. Davis Company, Philadelphia.

This work partakes of the character of an inquiry into the nature and cause of sexual impulse, and is based on elaborate investigations into the mode of life of past and contemporary savage races as a type of primitive man. It is only from a study of primitive sexual impulse that we can hope to gain accurate knowledge of perverted sexuality so frequently met in practice. The deeds of the "sadist" and the "rapist" form a dark shadow in the light of our boasted civilization, and their prophylaxis and cure are matters that should be close to every true physician's heart.

We have read the book with interest and profit ourselves, and can recommend it as a thoroughly scientific work. J. M. L.

